# Effects of Physical Activity Training in Patients with Alzheimer’s Dementia: Results of a Pilot RCT Study

**DOI:** 10.1371/journal.pone.0121478

**Published:** 2015-04-17

**Authors:** Vjera A. Holthoff, Kira Marschner, Maria Scharf, Julius Steding, Shirin Meyer, Rainer Koch, Markus Donix

**Affiliations:** 1 Department of Psychiatry and Psychotherapy, Division of Old Age Psychiatry and Cognitive Neuropsychiatry, Faculty of Medicine Carl Gustav Carus, Technische Universität Dresden, 01307, Dresden, Germany; 2 DZNE, German Center for Neurodegenerative Diseases, Dresden, Germany; University of Glasgow, UNITED KINGDOM

## Abstract

**Background:**

There is evidence that physical activity (PA) is of cognitive benefit to the ageing brain, but little is known on the effect in patients with Alzheimer’s disease (AD). The present pilot study assessed the effect of a home-based PA training on clinical symptoms, functional abilities, and caregiver burden after 12 and 24 weeks.

**Methods:**

In an RCT thirty patients (aged 72.4±4.3 years) with AD (MMSE: 20.6±6.5 points) and their family caregivers were allocated to a home-based 12-week PA intervention program or the usual care group. The program changed between passive, motor-assisted or active resistive leg training and changes in direction on a movement trainer in order to combine physical and cognitive stimuli.

**Results:**

Analysis of activities of daily living in the patients (ADCS ADL total score) revealed a significant group × time interaction effect (95% CI of the difference between both groups at T2: 5.01–10.51). The control group experienced decreases in ADL performance at week 12 and 24 whereas patients in the intervention group remained stable. Analyses of executive function and language ability revealed considerable effects for semantic word fluency with a group × time interaction (95% CI of the difference between both groups at T2: 0.18–4.02). Patients in the intervention group improved during the intervention and returned to initial performance at week 12 whereas the controls revealed continuous worsening. Analyses of reaction time, hand-eye quickness and attention revealed improvement only in the intervention group. Caregiver burden remained stable in the intervention group but worsened in the control group.

**Conclusions:**

This study suggests that PA in a home-based setting might be an effective and intrinsically attractive way to promote PA training in AD and modulate caregiver burden. The results demonstrate transfer benefits to ADL, cognitive and physical skill in patients with AD.

**Trial Registration:**

ClinicalTrials.gov NCT02196545

## Introduction

Animal studies have demonstrated physical exercise effects on brain function over the lifespan. Activity in an enriched environment stimulates the brain on a physical and cognitive level and has the potential to induce brain plasticity [1]. In humans growing evidence suggests that lifestyle factors have a significant impact on how well non-demented people age, and physical activity (PA) is one of the most important protective factors against cognitive decline [2]. However, only few studies have studied the effect of PA in patients already suffering from Alzheimer’s dementia (AD) [3,4]. Three pilot randomised controlled trials (RCTs) and one larger RCT were able to demonstrate significant cognitive benefits for AD patients and also on quality of life and depression. Study limitations included sample size, lack of information on the use of psychotropic medication [5], nursing home setting [6,7] and discrepancies in contact time [8]. Recent research has demonstrated that PA may induce neuroplastic changes in older age and therefore exert a protective effect against cognitive decline and that this may also occur in patients already suffering AD, thus inducing improvement of clinical symptoms [8,9].

In the study presented here we were interested in the effect of a specific home-based PA program performed on a movement trainer combining physical and basic cognitive stimuli and in the clinical impact on both, patients and caregivers.

Physical intervention that contrasts a monotonous bicycle ergometer training by providing training resistance level (passive, assisted, active) or direction (forward, reverse) changes may enrich a basic cycling intervention. Prior research has shown that intervention strategies targeting multiple factors separately are more effective than strategies focusing on single mechanisms or domains [10]. Anderson-Hanley et al. [11] showed superior effects (23% relative risk reduction for cognitive decline) of stationary cycling exercise among older adults if the cycling was performed in a virtual environment. The authors suggest that simultaneous cognitive and physical exercise has greater potential for preventing cognitive decline. However, it is important to highlight that our intervention design differs from others combining physical and cognitive stimuli. It remains unknown whether the ‘cognitive’ component complementing a physical intervention would require a specific level of complexity to elicit additional beneficial effects.

The decline in activities of daily living (ADL) in AD [12,13] is a source of considerable caregiver burden and socio-economic costs. Clinical care therefore specifically focusses on maintaining ADL functionality in AD patients. Two meta-analyses report that being physically active reduces the risk of progression of basic ADL disability in community dwelling adults [14] and in patients with dementia [15]. The primary outcome measure defined in our investigation therefore was performance in activities of daily living in the patients with secondary outcomes covering overall cognition, executive function and language control, attention, reaction time and hand-eye quickness as well as behavioural symptoms of dementia and self-reported caregiver burden. Putative long-term effects were studied in a post-treatment follow-up.

## Methods

The protocol for this trial and supporting CONSORT checklist are available as supporting information; see S1 CONSORT Checklist and S1 Protocol.

### Study population

The Ethics Committee of the University of Technology, Dresden, Germany, approved the study in May 2011 (EK 111032011). This study is a feasibility study and was aimed at testing the PA program chosen in a study population of patients suffering dementia, e.g. the patients’ ability to train on the movement trainer without the caregivers’ presence, the adherence to the program at a frequency expected to be necessary for a training effect. Furthermore, the purpose of the study was to estimate a recruitment rate of patients with AD and caregivers and the definition of a sample size necessary for a confirmatory study. At the time of submission of the study protocol, the Ethics Committee did not require registration for feasibility or proof of concept studies. The study was registered in ClinicalTrials.gov (NCT02196545) in July 2014 in preparation of a manuscript for publication of the data. The authors confirm that all ongoing and related trials for this intervention are registered. Patient and caregiver enrolment started in August 2011 and ended July 2013 and informed written consent was obtained by all participants.

A total of thirty patients with mild to moderate AD meeting NINCDS-ADRDA criteria aged 55 years or older and their family caregivers were recruited in the Memory Disorder Clinic, University Hospital Carl Gustav Carus, Dresden. We only recruited patients with early and moderate stage AD (CDR stage 1 and 2) who had the full capacity to consent. The capacity to consent was established in a clinical evaluation by an experienced and independent psychiatrist who was not involved in the study. All study participants were right-handed and underwent medical history evaluation and neuropsychological testing. The AD diagnoses was established by old age psychiatrists, based on their own patient evaluation and detailed neuropsychological testing administered by an experienced neuropsychologist including the following measures: Mini Mental State Examination (MMSE); Wechsler Memory Scale—Revised; Controlled Oral Word Association Test, letters F,A,S; California Verbal Learning Test. Structural MRI and laboratory testing results complemented the diagnostic procedures to rule out conditions that would have explained the dementia syndrome otherwise. Patients were reported to have a low habitual activity level by their caregiver, equalling less than 30 minutes time spent in activity by walking per day [16]. Patients were required to speak German as the dominant language (necessary for neuropsychological testing), a minimum of 8 years formal school education and a caregiver (e.g. spouse) living at home with the participant. Furthermore, patients entered the study if internal examination was free of contradictions to physical activity and if they were on a stable dose of pharmacological dementia treatment according to German guidelines (DGPPN) with acetycholinesterase inhibitor or memantin or combination for at least 6 months. Patients underwent physical and neurological examination and electrocardiogram. Participants with clinically relevant medical conditions, e.g. heart disease, hypertension or diabetes or a medication that could influence cognitive functioning (e.g. benzodiazepines, sleep aids, neuroleptics) were excluded from study participation. In addition exclusion criteria were history of alcohol or substance abuse, head trauma, psychiatric or neurological disorder preceding AD onset, or major systemic disease affecting brain function.

Patients were randomly allocated to the 12-week PA intervention program or to the usual care group. Participants were handed a pedometer worn for 7 days prior to and after completion of the study to document their usual daily activity level. Patients in the intervention group trained their lower body on a movement trainer (ReckMOTOmed) with a computer controlled and individually preassigned training flow. Caregivers were asked to choose a familiar chair prior to study begin. The movement trainer was positioned in front of the patient and PA training was performed from the comfort of that chair. We anticipated that using the familiar chair would encourage the patient’s participation and reassure the caregiver that PA would not cause adverse reactions, e.g. falls. Participants were required to train three times a week for 30 minutes at an individually chosen time with at least one day without training in between two training days. Times and dates of PA training were documented by the movement trainer computer system. Patients were considered to have successfully completed the intervention if they trained for at least 75% of the time required by the protocol. The program changed between passive, motor-assisted or active resistive training of the legs as well as changes in direction (forward, reverse) every 5 minutes. During a familiarization session prior to the study patients were asked to use the movement trainer and to respond to whether they felt that the training resistance level was appropriate (too easy, too high, just right). They started with level 1 and the level was increased as they made their choice. The movement trainer has 20 levels of motor resistance and the patients in the intervention group chose an activity level between 2 and 4. The level did not change over time. Caregivers were asked to act encouraging but to leave the room as soon as the patient started training. This design was chosen to control for social contact times between both groups. The movement trainer was removed from the patients’ homes after completion of the 12-week PA program. The control group received the same monthly clinical visits and a counselling by the treating physician, which included specific advice how to change inactive habits and increase the PA level. All participants underwent testing by a blinded psychologist at baseline, 12 and 24 weeks (at 10 a.m.). Measures included activities of daily living (ADL, ADCS ADL total score) and behavioural symptoms of dementia (NPI total score) and caregiver burden (NPI total burden score). Cognitive evaluation included the MMSE and measures of executive function and language ability applying the semantic and phonemic word fluency as measured by the CERAD [17] and the FAS-test [18]. The data was compared to age and education corrected normative data. Reaction time, hand-eye quickness and attention were measured using the reaction time ruler or FETZ-test [19]. The test determines how long it takes for a patient to respond to the dropping of a ruler with a length of 0.50m. The patient is asked to hold the ruler with his thumb and forefinger and to release the ruler while the investigator continues to hold it. The patient is instructed to catch the ruler as fast as he can, as soon as the investigator releases it. The number displayed on the ruler right over the patient’s thumb is noted. During the visits caregivers were interviewed and asked to report their experience with the training program and with leaving the patients alone during PA (intervention group) or their progress in daily PA (controls).

### Statistical methods

We performed univariate analyses between the intervention and control group on clinical and demographic variables using the Wilcoxon-Mann-Whitney U-test for continuous variables and the Fisher's exact test for categorical variables.

Mixed effect models were applied to measure the efficacy of the intervention for longitudinal data and to account for the repeated measurements across time. These models included parameters for the treatment group (intervention and control group), for the three time points (T0- baseline, T1—3months later or after completion of the intervention and T2- 3 month follow-up) and for time x treatment interaction. Analyses included the measures for each outcome variable at T0 as covariable.

An autoregressive first order covariance structure was used for repeated measures. Model based estimations of the means and differences of the means and of their confidence intervals adjusted for overall mean baseline values were computed of all outcome variables. Formal Tukey adjusted multiple statistical tests were used for the primary variable ADL only. All analyses were performed with SAS 9.3. (SAS Institute Inc., Cary, North Carolina).

## Results

**Fig 1:**
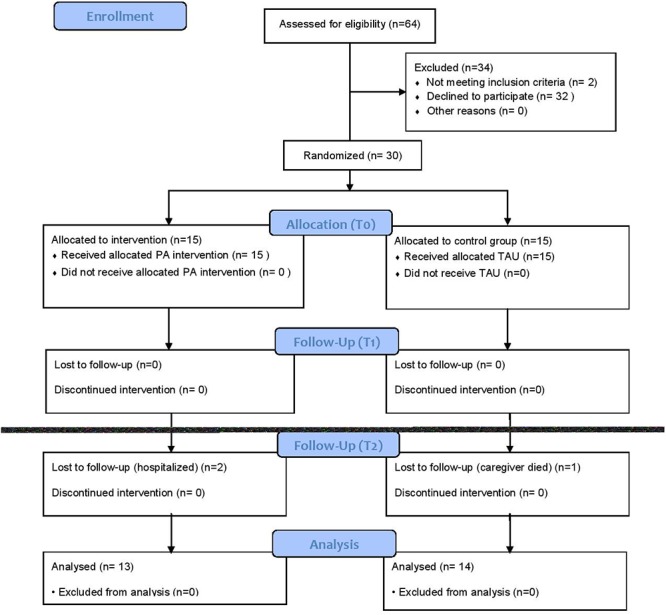
Consort Flow Chart.

Thirty patients with AD were randomized to either the intervention (n = 15) or control group (n = 15). A total of 64 patients were eligible and contacted by the physicians of the memory disorder clinic of the university hospital. 32 patients and caregivers declined participation as they doubted that the patients would adhere to the study protocol as often as required or because they wanted to travel freely during the three months to follow. Two patients were excluded as they had progressed to severe dementia since first screening for the study (Fig 1). The restricted availability of the movement trainers and the allocation to each participant for a period of 3 months made the recruitment period last from August 2011 to July 2013.

All participants in the intervention group successfully completed the study (minimum of 27 training units of 30 minutes over 12 weeks and a frequency of 3/week) and all participants in the control group completed the first follow-up 12 weeks after study inclusion. One patient in the control group and 2 patients in the intervention group were not available for long-term follow-up after 6 months (hospitalization and death of the caregiver). Demographic and clinical characteristics are reported in Table 1. At the time of the study all participants were on a stable treatment for AD (acetylcholinesterase inhibitor, memantin or combination) for at least 6 months and did not receive any other medication that could influence cognitive functioning (e.g. benzodiazepines, sleep aids, neuroleptics).

**Table 1:** Demographic and clinical characteristics.

	Intervention group		Control group		
	N = 15		N = 15		*p
**Age, mean (SD) in years**	72.40	(4.34)	70.67	(5.41)	0.34
**Female, N (%)**	8	(53.3)	7	(48.7)	0.71
**Age of onset, mean (SD) in years**	68.27	4.98	67.87	6.35	0.85
**Education, mean (SD) in years**	12.33	2.13	13.13	2.70	0.07
**Number of steps, N (SD)**	5818	4180	6662	4986	0.62
**BMI, N (SD)**	23.43	2.75	24.12	4.06	0.59

*p-values (chi-square and t test)

No considerable differences between both groups were noted in the baseline data for demographic and clinical measures. Patients trained at a relatively uniform level with respect to training frequency and time.

**Fig 2:**
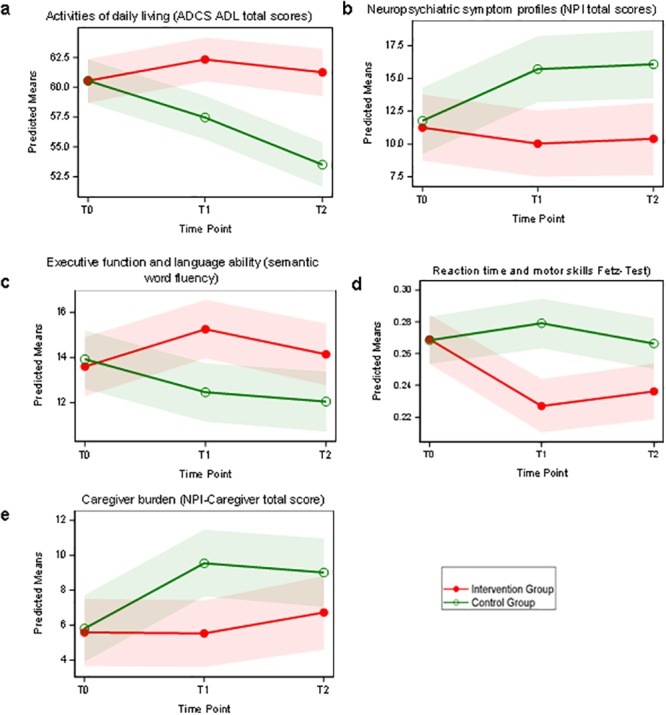
a-e. Effects of physical activity on clinical performance. This figure shows the effects of physical activity on the patients when compared to the control group for the three time points (T0- baseline, T1–3 months later or after completion of the intervention and T2- 3 month follow-up). Activities of daily living (ADCS ADL total scores): patients in the control group experienced significant decreases in their performance over 12 weeks and at the 3 month follow-up whereas patients in the intervention group remained stable during the study period and follow-up (Fig 2a). Neuropsychiatric symptom profiles (NPI total scores): controls suffered a considerable increase in behavioural changes over 24 weeks whereas patients in the intervention group remained stable over 24 weeks (Fig 2b). Executive function and language ability: patients in the intervention group improved during the intervention period and returned to initial performance after completion but without revealing the continuous worsening over 24 weeks demonstrated in the controls (Fig 2c). Reaction time, hand-eye quickness and attention (FETZ-test or Ruler Drop Test): only patients in the intervention group improved their performance during the study period (Fig 2d). Caregiver burden (NPI): burden increased in the control group during the first 3 months whereas caregiver burden remained stable in the intervention group during the study period (Fig 2e).

Longitudinal analysis of the patients’ ADL (ADCS ADL total scores, the primary outcome) revealed a significant group × time interaction effect (95% CI of the difference between both groups at T2: 5.01–10.51). Patients in the control group experienced considerable decreases in their performance in ADL over 12 weeks and at the 3 month follow-up whereas patients in the intervention group remained stable during the study period and follow-up (Fig 2a, Table 2). Neuropsychiatric symptom profiles as measured by NPI total scores showed a considerable group × time interaction effect (95% CI of the difference between both groups at T2: 1.83–9.55). Controls suffered a considerable increase in behavioural changes over 24 weeks whereas patients in the intervention group remained stable over 24 weeks (Fig 2b, Table 2). Analyses of the specific behavioural symptoms (NPI subscores) showed that depression (main effect of group estimated to 1.12 with s.e. = 0.51) and anxiety (main effect group x time interaction estimated to 1.50 with s.e. = 0.67) revealed clinically relevant worsening in the controls. Analyses of executive function and language ability revealed considerable effects for semantic word fluency with a considerable group × time interaction (95%CI of the difference between both groups at T2: 0.18–4.02) (Fig 2c, Table 2). Patients in the intervention group considerably improved during the intervention period and returned to initial performance after completion but without revealing the continuous worsening over 24 weeks demonstrated in the controls. The measure for global cognitive function as measured by MMSE (20.6±6.5 points) did not reach significance (F = 0.77, df = 2, 53, p = 0.4659).

**Table 2:** Model predicted means and their differences.

Variable	Intervention group mean (s.e.)	Control group mean (s.e.)	difference of means (95%CI)	residual variance (covariance of neighbouring time points)
Activities of daily living (ADCS ADL total score)	T0: 60.55 (0.91)	T0: 60.53 (0.91)	T0: 0.02 (-2.57–2.61)	12.45
	T1: 62.35 (0.91)	T1: 57.47 (0.91)	T1: 4.89 (2.30–7.48)	(0.50)
	T2: 61.26 (1.00)	T2: 53.50 (0.94)	T2: 7.76 (5.01–10.51)^a)^	
Neuropsychiatric symptom profiles (NPI total score)	T0: 11.25 (1.26)	T0: 11.77 (1.26)	T0: -0.51 (-4.15–3.10)	22.91 (0.45)
	T1: 10.05 (1.26)	T1: 15.71 (1.26)	T1: -5.66 (-9.28 –-2.03)	
	T2: 10.40 (1.38)	T2: 16.09 (1.29)	T2: -5.69 (-9.55 –-1.83)^a)^	
Executive function and language ability (semantic word fluency, number of words)	T0: 13.60 (0.65)	T0: 13.92 (0.65)	T0: -0.32 (-2.17–1.53)	6.21
	T1: 15.27 (0.65)	T1: 12.46 (0.65)	T1: 2.81 (0.96–4.66)	(0.39)
	T2: 14.15 (0.69)	T2: 12.05 (0.67)	T2: 2.10 (0.18–4.02)^a)^	
Reaction time and motor skills (Fetz-test, meter)	T0: 0.27 (0.01)	T0: 0.27 (0.01)	T0: 0.001 (-0.02–0.02)	0.00077
	T1: 0.23 (0.01)	T1: 0.28 (0.01)	T1: -0.052 (-0.08 –-0.03)	(0.1822)
	T2: 0.24 (0.01)	T2: 0.27 (0.01)	T2: -0.03 (-0.05 –-0.01)^a)^	
Caregiver burden (NPI-caregiver total score)	T0: 5.58 (0.96)	T0: 5.82 (0.96)	T0: -0.24 (-2.97–2.49)	13.50
	T1: 5.51 (0.96)	T1: 9.55 (0.96)	T1: -4.04 (-6.77 –-1.31)	(0.39)
	T2: 6.71 (1.05)	T2: 9.00 (0.98)	T2: -2.29 (-5.20–0.62)	
MMSE (total score)	T0: 22.05 (0.54)	T0: 21.95 (0.54)	T0: 0.10 (-1.43–1.63)	4.30
	T1: 21.99 (0.54)	T1: 21.28 (0.54)	T1: 0.70 (-0.83–2.23)	(0.35)
	T2: 22.11 (0.57)	T2: 20.72 (0.55)	T2: 1.39 (-0.21–2.98)	

^**a**)^ significant at T2 (p<0.05); T0: baseline, T1: 3 months later or after completion of the intervention, T2: 3 month follow-up.; Results of the FETZ-test are given in meters, the lower the number the shorter the reaction time.

Analyses of reaction time, hand-eye quickness and attention (Ruler Drop Test) revealed a considerable group x time interaction (95%CI of the difference between both groups at T2: 0.006–0.054) and indicates that only patients in the intervention group improved their performance during the study period (Fig 2d, Table 2). Caregiver burden was measured using the NPI and analyses revealed a considerable group × time interaction (95%CI of the difference between both groups at T2: 0.62 –-5.20). Caregiver burden in the control group considerably increased during the first 3 months whereas caregiver burden remained stable in the intervention group during the study period (Fig 2e, Table 2). There was no difference in daily activities as measured by the pedometers worn 7 days prior to and following completion of the study within and between groups.

## Discussion

We found that the PA training program presented here is of practical and clinical relevance to the treatment of patients with AD and to caregiver burden. The flexible training schedule and accessibility of the movement trainer associated with the home-based setting were beneficial: exercise adherence was excellent and measures of differing time and dates for PA training obtained in this study were in line with the clinical experience how difficult it is to overcome fluctuations in AD patients’ activity levels. The activity trainer used made it possible for the patients to be seated on a familiar chair and may have contributed to the fact that caregivers were not reluctant to leave the patients alone during PA training as revealed by the questionnaire reports. Patients in the intervention group did not improve their activity level in steps per day at follow-up, which highlights the specific attractiveness of PA training at home to increase the habitual activity level per week. Benefits in the context of a progressing dementing illness such as AD are a lack of significant decline during the intervention period, or long-term effects at follow-up measures if performance remains significantly better when compared to controls. In fact, the results demonstrated considerable benefits of PA training on cognitive, behavioural and motor function, including of these measures ADL, executive function and language ability, neuropsychiatric symptoms, reaction time, hand-eye quickness and attention in patients, and reduction of caregiver burden. Analyses demonstrated long-term effects three months after completion of the intervention on ADL and behavioural symptoms. Executive function is the key cognitive resource responsible for self-regulation of behaviours including the ability to plan, initiate, sequence and monitor [20]. In AD executive dysfunction is a prominent clinical symptom directly affecting the patient’s capacity in activities of daily living [12,13]. Previous studies reported that aerobic exercise leads to an increase in executive function performance in non-demented elderly with cognitive impairment [21,22,23,24,25]. The beneficial effect of PA on the aging brain or in dementia [3] is not well explained, but animal studies have revealed activation of adult neurogenesis [1] or increases in plasma levels of the neuroplasticity associated brain-derived neurotrophic factor [26]. The effect of PA on clinical performance in AD may therefore rely on improvement of brain functionality. Furthermore, data revealed that patient training had a considerable effect on caregiver burden as it remained stable during the intervention period whereas it considerably increased in the control group. A number of studies have demonstrated that behavioural symptoms in AD are a source of distress and burden for family and professional caregivers, and are associated with more rapid institutionalization for patients with AD [27]. The strengths of this pilot RCT are the home-based design, PA intervention combining physical and cognitive stimuli, and a design aiming at minimizing social interaction effects between both study groups. Given the relatively brief intervention period we did not change the individual resistance level over time. In contrast to investigating cognitive changes following exercise at an individual performance limit in a laboratory setting (e.g., while monitoring heart rate and other vital parameters) our approach was to administer an exercise that would require moderate physical resources and would therefore be acceptable for everyday use among demented patients in their own homes without the help of a trainer or instructor. Important limitations are the sample size and the lack of an active control group. As the caregivers could not be blinded for the condition, the measures of neuropsychiatric symptoms (NPI total score) and caregiver burden (NPI caregiver burden) may be biased. Furthermore, we did not specifically investigate whether the additional stimuli (changes in passive, assisted, active training and cycling direction) complementing the physical intervention would be associated with independent effects on outcome parameters. Future studies may help determine which stimuli during physical exercise may not only modulate alertness and intervention adherence but also represent cognitive components with unique effects on behavioural changes.

Based on the results obtained in this pilot study we would expect a recruitment rate of 1.25 patients per month in a confirmatory study. The estimation of the sample size was based on three primary outcome variables we suggest including MMSE with the lowest effect in our pilot study (power of 80% for an effect of 1.2 score points in MMSE). For a confirmatory study with an intervention and a control group the choice of three primary outcome variables including the measures for ADL (ADL-ADCS), overall cognition (MMSE) and executive function (semantic word fluency) would necessitate a sample size of 92 patients in each group at a global significance level of 0.05 (Bonferroni adjusted over all three primary hypotheses).

In summary, this study suggests that PA on a movement trainer in a home-based setting, which combines cognitive and physical demands in an intrinsically attractive activity, might be an effective way to promote PA training in AD and modulate caregiver burden. The results are encouraging and demonstrate transfer benefits to ADL, cognitive and physical skills that determine functional abilities in patients with AD.

## Supporting Information

S1 CONSORT Checklist CONSORT Checklist. (PDF)

S1 Protocol Trial Protocol. (PDF)

S1 Dataset This dataset contains all time dependent MOTODEM variables. (SAV)

S2 Dataset This dataset contains all time independent MOTODEM variables. (SAV)
