# Supplementary material for: Effects of Physical Activity Training in Patients with Alzheimer’s Dementia: Results of a Pilot RCT Study
Source: PLoS One. 2015 Apr 17;10(4):e0121478. doi: 10.1371/journal.pone.0121478 (PMC4401690; doi:10.1371/journal.pone.0121478)
Supplement: S1 Protocol — (PDF) [file pone.0121478.s002.pdf]

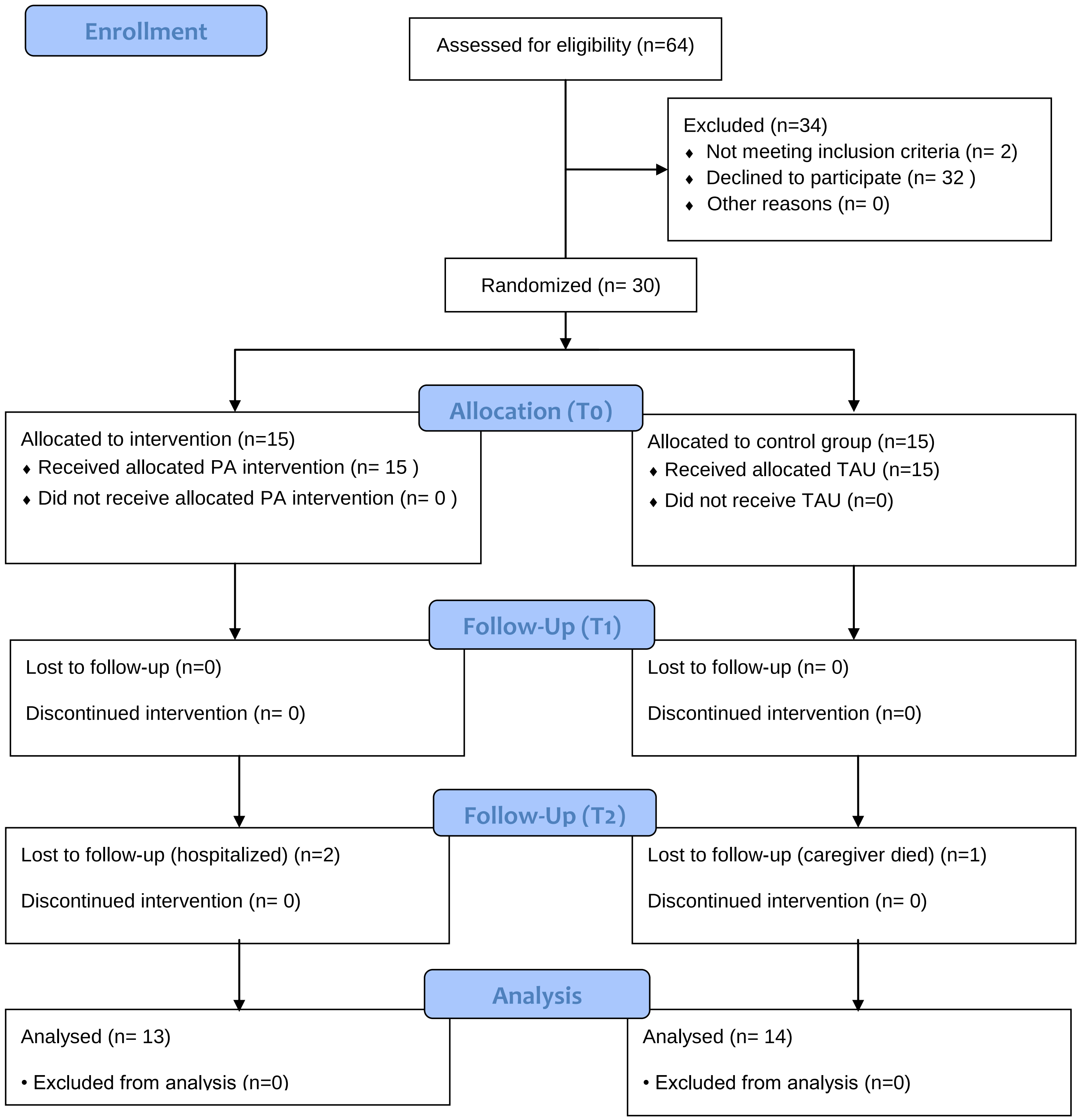
Enrollment
Assessed for eligibility (n=64)
Excluded (n=34)
♦ Not meeting inclusion criteria (n= 2)
♦ Declined to participate (n= 32)
♦ Other reasons (n= 0)
Randomized (n= 30)
Allocation (T0)
Allocated to intervention (n=15)
♦ Received allocated PA intervention (n= 15)
♦ Did not receive allocated PA intervention (n= 0)
Allocated to control group (n=15)
♦ Received allocated TAU (n=15)
♦ Did not receive TAU (n=0)
Follow-Up (T1)
Lost to follow-up (n=0)
Discontinued intervention (n= 0)
Lost to follow-up (n= 0)
Discontinued intervention (n=0)
Follow-Up (T2)
Lost to follow-up (hospitalized) (n=2)
Discontinued intervention (n= 0)
Lost to follow-up (caregiver died) (n=1)
Discontinued intervention (n= 0)
Analysis
Analysed (n= 13)
• Excluded from analysis (n=0)
Analysed (n= 14)
• Excluded from analysis (n=0)
